# Dataset representing growth performance, nutritional assay and biochemical profile of *Oscillatoria spp*.

**DOI:** 10.1016/j.dib.2024.110255

**Published:** 2024-02-28

**Authors:** Jannatul Nayeem, Proma Dey, Sumit Kanti Dey, Rezaul Karim, Mohammed Ayoun, Abed Hasan Tuser, Nusrat Zaman Zemi, Helena Khatoon

**Affiliations:** a Department of Aquaculture, Chattogram Veterinary and Animal Sciences University, Chattogram 4225, Bangladesh; b Institute of Technology Transfer and Innovation, Bangladesh Council of Scientific and Industrial Research, Dr Qudrate-I-Khuda Road, Dhaka 1205, Bangladesh

**Keywords:** Cyanobacteria, *Oscillatoria*, Isolation, Growth curve, Nutritional composition, Fatty acid, Amino acid

## Abstract

Cyanobacteria are regarded as vital constituents of aquatic ecosystems which recently become viable option for bioremediation since it can remove contaminants from polluted water. They possess intriguing metabolic properties and exhibit differential growth patterns. This study elucidates the isolation and identification of two marine and two freshwater indigenous *Oscillatoria* spp., their growth performance, nutritional composition along with intricate biochemical profiles. Agar streak plate method was used for the isolation, growth curve was determined through chlorophyll content and optical density. Freshwater and marine *Oscillatoria* spp. were mass cultured in commercial Bold Basal Media and Conway media respectively. Wet biomass was harvested through centrifugation at the early stationary phase of their respective growth curve and oven-dried at 40 °C to determine the nutritional and biochemical profiles. *Oscillatoria* sp. 2 displayed significantly higher (*p* ˂ 0.05) chlorophyll-a (22.72 ± 0.04 µg/mL) and OD value (1.87 ± 0.03) in the stationary phase (9th to 11th day) than the other species. Crude protein contents (%) varied from 21.56 ± 0.09 to 56.97 ± 0.03. Crude lipid (%) ranged from 9.07 ± 0.07 to 17.13 ± 0.13 and Crude fiber content (%) showed the range from 7.49 ± 0.15 to 17.04 ± 0.08. Fatty acid and amino acid were also found variable among the species. Present study will contribute to the meticulous selection and characterization of *Oscillatoria* sp. to utilize it in the rigorous scientific investigations and diverse commercial applications.

Specifications Table

**Table:** 

Subject	Food Science, Aquatic Science
Specific subject area	Growth performance, nutritional assay and biochemical profile of isolated indigenous *Oscillatoria* spp.
Data format	Raw and analyzed primary data
Type of data	Picture, Graph and Table
Data collection	Data on isolation were attained by performing agar streak plate method. Identification was performed through cellular, colony morphology and computer based light microscopic observation. Data on physical parameters of sampling site water including temperature, dissolve oxygen, pH were collected with glass thermometer, dissolved oxygen meter (DO-5509, Lutron) and a portable pH meter (pHep-HI98107, HANNA, Romania) respectively. Chemical parameters (TAN, SRP and NO_2__—_N) were determined following the spectrophotometric methods of Parsons et al. (1984). Chlorophyll and optical density data were assessed spectrophotometrically to determine the growth patterns. For fatty acids: Gas Chromatography Mass Spectrophotometry GCMS analysis of saturated fatty acids, monounsaturated fatty acids, polyunsaturated fatty acids, omega 3 and omega 6 fatty acids. For amino acids: SYKAM amino acid analysis of *Oscillatoria* spp.; essential amino acids, non-essential amino acids. The acquired data were further analyzed through MS Excel and IBM SPSS (v. 26.0) software.
Data source location	Kaptai Lake, Rangamati (22°64′ N, 92°19′ E); Halda River, Chattogram (22°51′ N, 91°84′ E); Maheshkhali channel, Cox's Bazar (21°31′ N, 91°59′ E) and Naf River estuary, Teknaf; Cox's Bazar (20° 47′ N, 92° 28′ E); Bangladesh Microalgae Research Corner and Disease and Microbiology Laboratory, Department of Aquaculture, Faculty of Fisheries, Chattogram Veterinary and Animal Sciences University (CVASU), Khulshi-4225, Chattogram, Bangladesh
Data accessibility	Data are available with this article and also at Repository name: Mendeley Data Data identification number: Direct URL to data: https://data.mendeley.com/datasets/v9xwnvn39p/1

## 1 Value of the Data

• The findings on isolation enumerate the diversity, water quality, ecology and unexplored characterization and utilization potentials of the *Oscillatoria* species.
• Findings of the growth phases outline the harvesting time of *Oscillatoria* sp. and also provide insights of the ecological roles of *Oscillatoria* sp.; assist to predict algal blooms, indicate bioremediation strategies, aid in screening and selecting species for commercial sector and fosters conversations on climate change and aquaculture management.
• Nutritional and biochemical data contribute to the comprehensive knowledge of their metabolic mechanisms, functional and sustainable biofuel production potentialities. These data are valuable in advancing the aquaculture and nutraceutical sectors, assisting ecological assessments, and initiating innovative eco-friendly biological research.

## 2 Background

Cyanobacteria are primitive Gram-negative prokaryotes possess several distinctive characteristics, including oxygenic photosynthesis, high biomass production, adaptability to non-arable lands and diverse water sources (even wastewaters), the generation of valuable by-products and biofuels, soil fertility enhancement, and the reduction of greenhouse gas emissions. These combined attributes position these bio-agents as invaluable resources for sustainable development [2]. Cyanobacteria are regarded as renewable resource with diverse application potentialities in different sectors [3]. Cyanobacteria have garnered global attention for their potentialities in mariculture, food production, animal feed, fuel generation, fertilizer development, colorant production, and the synthesis of diverse secondary metabolites including vitamins, toxins, enzymes, pharmaceuticals, pharmacological agents, and pollution control [4]. However, only a limited number of cyanobacterial strains, including *Spirulina* sp., have been extensively characterized and commercially exploited. There's a crucial need for foundational research to explore new cyanobacterial strains that offer high-value products. Thus, this study aimed to isolate and characterized different *Oscillatoria* sp. to properly exploit them in different commercial sector (Tables 1 and 2).

**Table 1:** Physicochemical parameters of the sample water (mean±SE) gathered from several freshwater and marine water sampling sites in Bangladesh.

Parameters	Halda river	Kaptai lake	Naf River estuary	Maheshkhali channel
Temperature (°C)	30.03 ± 0.09^b^	31.03 ± 0.58^b^	33.00 ± 0.15^a^	31.40 ± 0.06^b^
DO (mg/L)	7.90 ± 0.06^b^	7.03 ± 0.09^d^	10.03 ± 0.09^a^	7.40 ± 0.06^c^
pH	8.10 ± 0.02^b^	8.30 ± 0.03^ab^	8.50 ± 0.10^a^	7.50 ± 0.11^c^
Salinity (ppt)	0.00 ± 0.00^c^	0.00 ± 0.00^c^	15.00 ± 0.12^b^	30.00 ± 0.00^a^
Total Ammonia nitrogen (TAN) (mg/L)	0.004 ± 0.001^b^	0.008 ± 0.001^a^	0.003 ± 0.00^b^	0.002 ± 0.001^b^
Soluble reactive phosphate (mg/L)	0.046 ± 0.003^a^	0.037 ± 0.003^a^	0.039 ± 0.003^a^	0.022 ± 0.002^b^
Nitrite-Nitrogen (mg/L)	0.023 ± 0.001^c^	0.052 ± 0.002^b^	0.085 ± 0.000^a^	0.086 ± 0.002^a^

**Table 2:** Morphological properties of *Oscillatoria* species isolated from different sampling sites in Bangladesh.

Cyanobacteria species	Characteristics
Oscillatoria sp. 1	➢ Planktonic, cylindrical, thread like structures which appeared in blue-green color. ➢ Species consists of a series of cells forming unbranched filaments or trichomes. ➢ Constricted at cross walls ➢ Cells are 5–7 µm long and 52 µm wide
Oscillatoria sp. 2	➢ Filamentous, barrel-shaped straight structures with slight bent edges. ➢ Thallus are long and comprises dark blue-green color to blackish blue-green color. ➢ Cells are solitary or in clusters and constricted at cross walls. ➢ Cells are 6–8 µm long and 50 µm wide
*Oscillatoria* sp. 3	➢ It contains Clusters like filaments and cells are cylindrical with tapering at the outer edges. ➢ Thallus are short and coiled and trichomes appeared as bright blue-green color. ➢ Trichomes are straight or slightly coiled, motile ➢ Cells are 3–4 µm in length and 47 µm in width
*Oscillatoria* sp. 4	➢ Filamentous, thallus is long and visible in dark blue-green color. ➢ Trichomes are straight to slightly curved with bent outer edges ➢ Trichromes are rarely solitary and motile in nature ➢ Cells are 6–8 µm in length and 51 µm width

## 3 Data Description

**Fig. 1:**
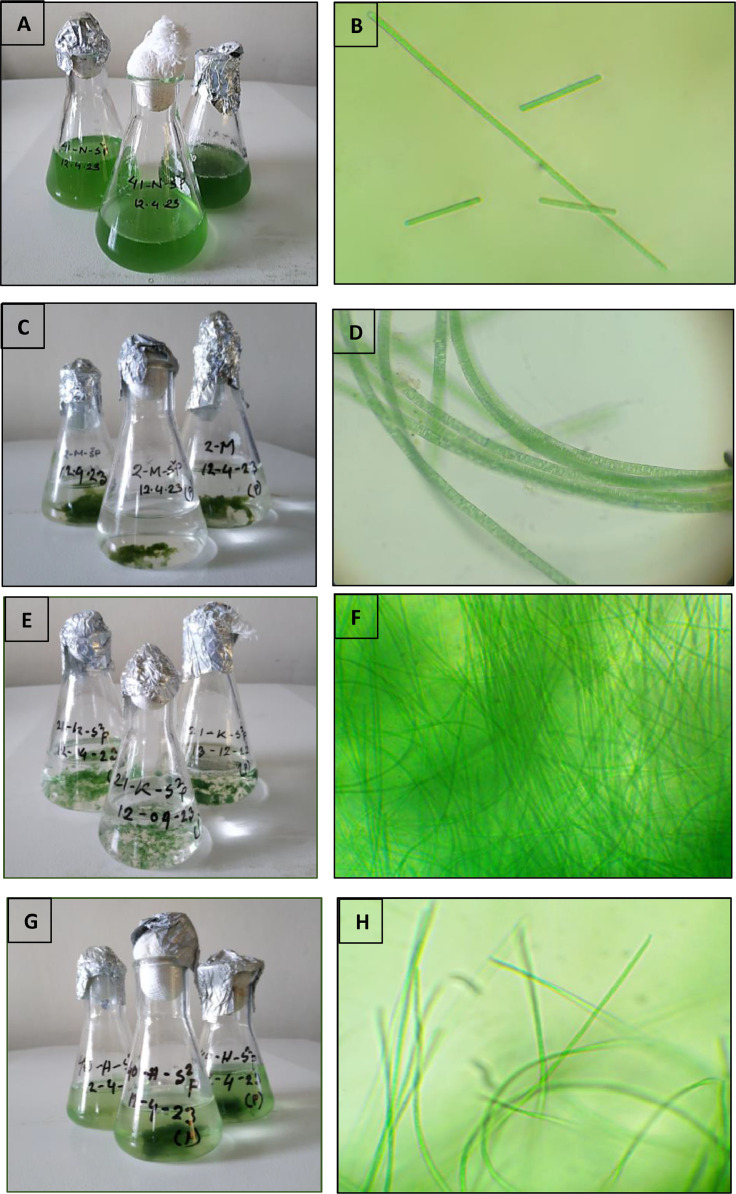
Colony structure and light microscopic pictures of isolated *Oscillatoria* spp.; Species 1 (A-Colony structure, B- microscopic view); Species 2 (C—Colony structure, d- microscopic view); Species 3 (E-Colony structure, F- microscopic view); Species 4 (G-Colony structure, H- microscopic view).

Four species of *Oscillatoria* were isolated and presented in this dataset along with their growth curve, nutritional composition, fatty acid and amino acid profile [1]. Marine *Oscillatoria* such as *Oscillatoria* sp. 1 (A, B) and *Oscillatoria* sp. 2 (C, D) were isolated from Naf River estuary and Maheshkhali channel respectively. Freshwater *Oscillatoria* including *Oscillatoria* sp. 3 (E, F) and *Oscillatoria* sp. 4 (G, H) were isolated from Kaptai lake and Halda river respectively (Fig. 1).

**Fig. 2:**
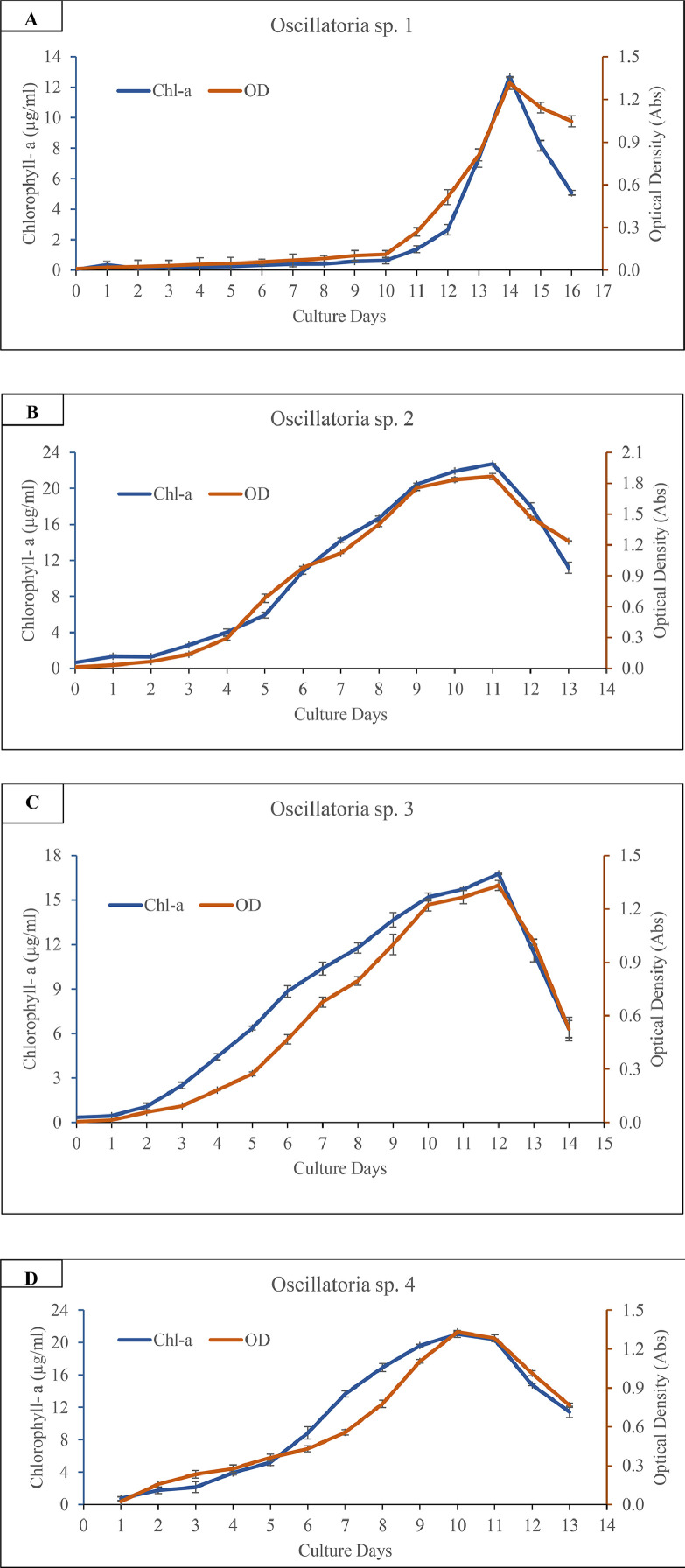
Growth curves of isolated *Oscillatoria* sp. 1 (A), *Oscillatoria* sp. 2 (B), *Oscillatoria* sp. 3 (C) and *Oscillatoria* sp. 4 (D) in terms of chlorophyll-a content (µg/mL) and optical density (Absorbance) Values are means of the triplicates with standard error. Chl-a and OD represent chlorophyll-a content, and optical density respectively.

**Fig. 3:**
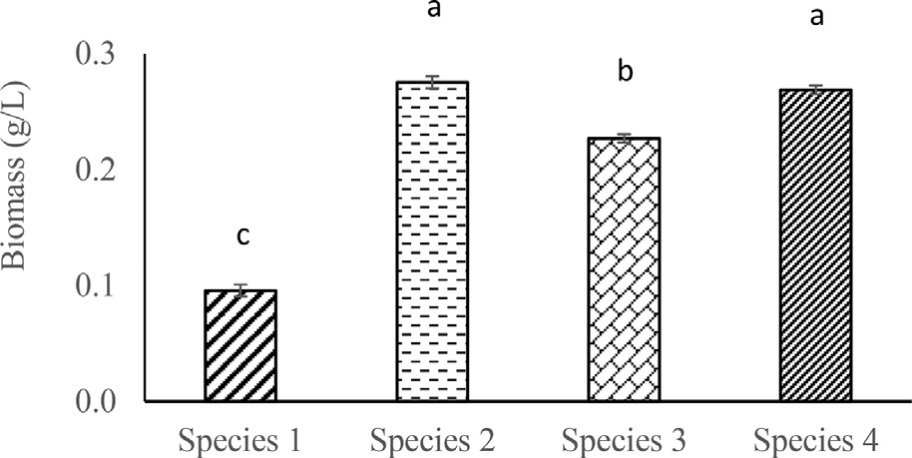
Dried biomass (g/L) of *Oscillatoria* spp. Values are average of the triplicates with standard error bar. Significant variations among the species (*p* < 0.05) are denoted by values in each category with a different letter.

Differential growth phases of each *Oscillatoria* spp. were observed and found significantly variable (*p* ˂ 0.05). Growth phases were determined through chlorophyll-a content and optical density of the respective species. Fig. 2 illustrated the chlorophyll content and optical density of each of the four species as a function of cultivation time. *Oscillatoria* sp. 2 displayed significantly higher (*p* ˂ 0.05) chlorophyll-a (22.72 ± 0.04 µg/mL) and OD value (1.87 ± 0.03) on 11th day compared to other species. Fig. 3 displayed the significant (*p* < 0.05) variations of dried biomass (g/L) among the *Oscillatoria* spp.

**Fig. 4:**
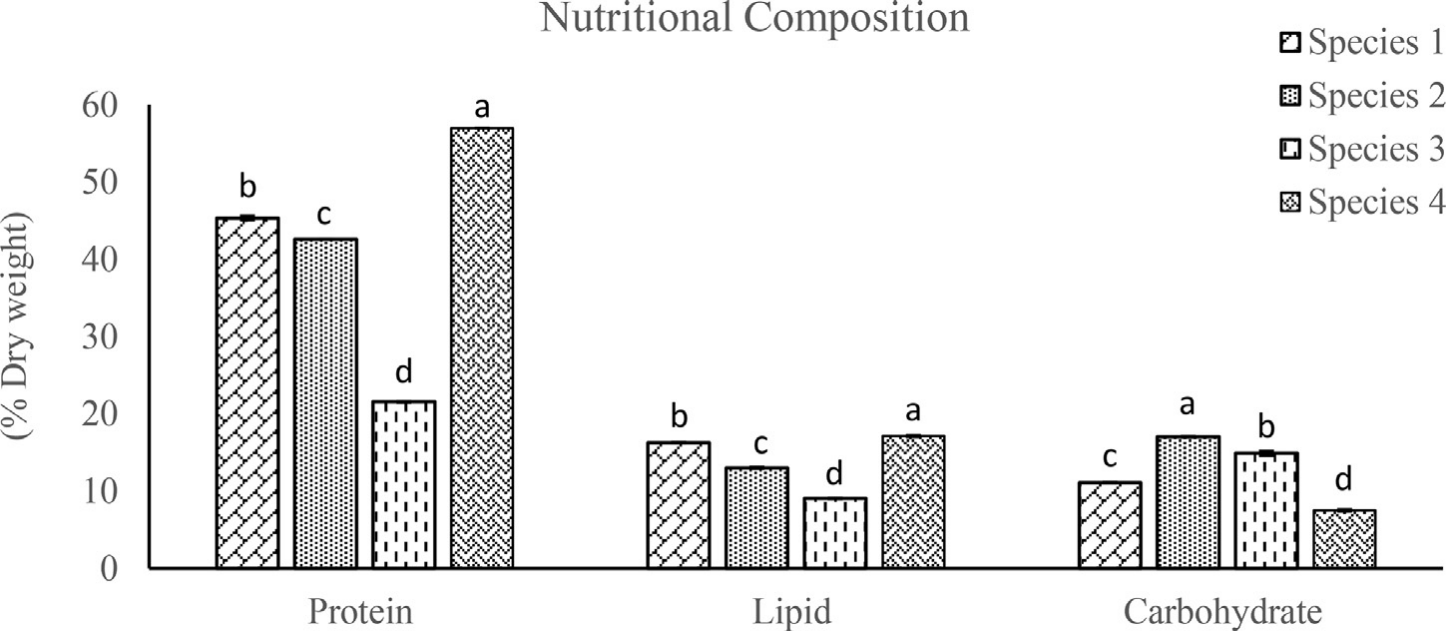
Nutritional composition including protein content (% dry weight) (mean ± SE), lipid content (% dry weight) (mean ± SE) and carbohydrate content (% dry weight) (mean ± SE) of *Oscillatoria* spp. Significant variations among the species (*p* < 0.05) are indicated by values in each series with a distinct letter.

The significant variations (*p* < 0.05) of the nutritional composition of *Oscillatoria* spp. are depicted in Fig. 4 where the percentages of crude protein, lipid and carbohydrate content are estimated from the dried biomass. Crude protein contents varied from 21.56 ± 0.09 to 56.97 ± 0.03, Crude lipid (%) ranged from 9.07 ± 0.07 to 17.13 ± 0.13 and crude carbohydrate content (%) showed the range from 7.49 ± 0.15 to 17.04 ± 0.08.

**Fig. 5:**
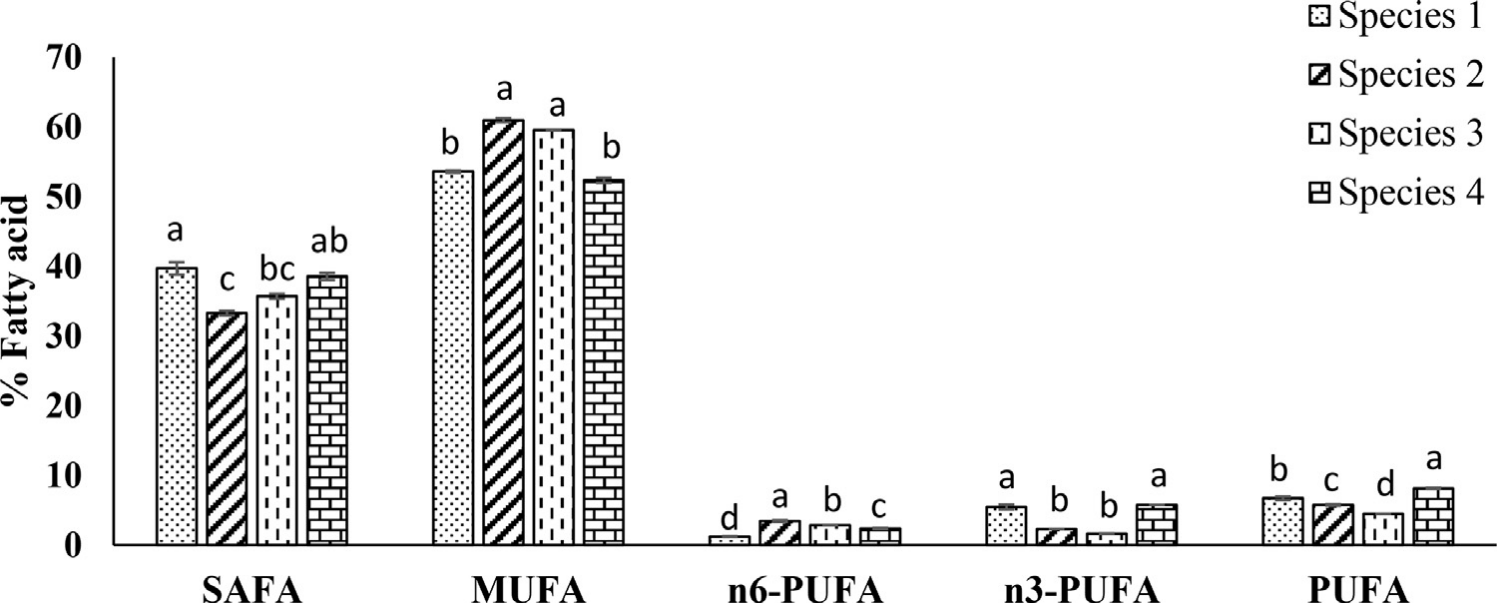
Fatty acid content (% total) of isolated *Oscillatoria* spp. Values are the average of duplicates with standard error (mean ± SE). Significant variations among the species (*p* < 0.05) are denoted by values in each series with a distinct letter. **SAFA**: Saturated Fatty Acids, **MUFA**: Monounsaturated atty acids, **n6-PUFA**: ω−6 polyunsaturated fatty acids, **n3-PUFA**: ω−3 polyunsaturated fatty acids, **PUFA**: Polyunsaturated fatty acids.

**Fig. 6:**
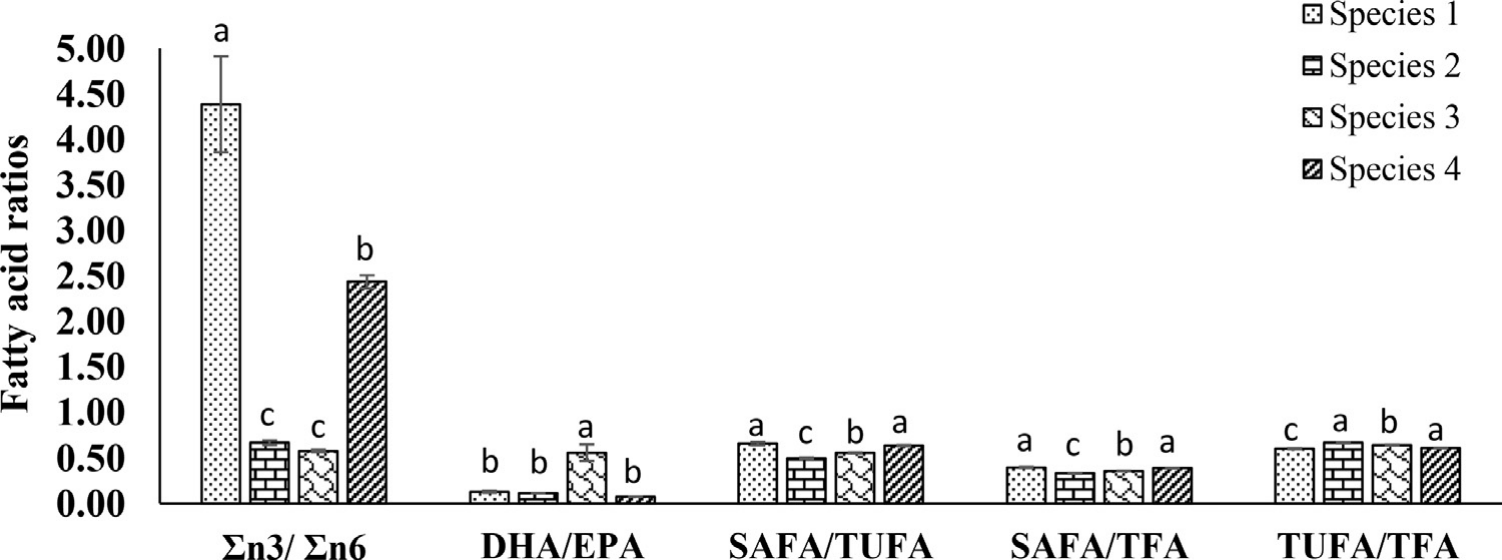
Fatty acid content ratios of isolated *Oscillatoria* spp. Values are the average of duplicates with standard error (SE = σ/√n). Distinct letter used in each series enumerate significant variations among the species (*p* < 0.05). **n3-PUFA**: ω−3 polyunsaturated fatty acids, **n6-PUFA**: ω−6 polyunsaturated fatty acids, **DHA**: Docosahexaenoic acid, **EPA:** Eicosapentaenoic acid, **TUFA**: Total unsaturated fatty acids, **TFA**: Total fatty acids.

Variations of the fatty acids (mean ± SE) are demonstrated in Table 3. Saturated fatty acids, monounsaturated fatty acids, poly unsaturated fatty acids were found significantly variable among the species (Fig. 5). Ratios of fatty acid content were also statistically significant (*p* < 0.05) among *Oscillatoria* spp. (Fig. 6).

**Table 3:** Fatty acids content (% total) of different *Oscillatoria* species expressed as mean of the duplicates with standard error (mean ± SE).

Carbon	Fatty Acid Methyl Esters	*Oscillatoria*			
		Species 1	Species 2	Species 3	Species 4
		
		Amount (%)			
Saturated Fatty Acid (SAFA)					
C8:0	Methyl Octanoate	0.48 ± 0.05	0.05 ± 0.00	0.03 ± 0.00	0.02 ± 0.00
C10:0	Methyl Decanoate	0.46 ± 0.01	4.48 ± 0.20	1.16 ± 0.15	3.57 ± 0.02
C12:0	Methyl Laurate	3.30 ± 0.06	0.04 ± 0.00	2.02 ± 0.00	2.23 ± 0.00
C13:0	Methyl Tridecanoate	0.27 ± 0.01	2.30 ± 0.09	1.89 ± 0.03	5.31 ± 0.00
C14:0	Methyl Myristate	0.06 ± 0.00	3.97 ± 0.32	2.74 ± 0.22	5.18 ± 0.49
C16:0	Methyl Palmitate	14.82 ± 0.18	7.34 ± 0.37	11.83 ± 0.46	10.37 ± 0.14
C18:0	Methyl Stearate	13.85 ± 0.70	8.14 ± 0.03	8.03 ± 0.40	8.59 ± 0.04
C20:0	Methyl Arachidate	1.36 ± 0.09	4.84 ± 0.02	3.49 ± 0.36	0.59 ± 0.00
C17:0	Methyl Heptadecanoate	0.97 ± 0.00	0.04 ± 0.00	1.92 ± 0.98	1.55 ± 0.04
C21:0	Methyl Heneicosanoate	0.22 ± 0.02	0.57 ± 0.09	0.82 ± 0.12	0.27 ± 0.02
C22:0	Methyl Behenate	3.91 ± 0.05	1.51 ± 0.04	1.76 ± 0.06	0.89 ± 0.01
C23:0	Methyl Tricosanoate	ND ± ND	ND ± ND	ND ± ND	ND ± ND
C24:0	Methyl Lignocerate	ND ± ND	ND ± ND	ND ± ND	ND ± ND
**Mono Unsaturated Fatty Acid (MUFA)**					
C16:1	Methyl Palmitoleate	16.43 ± 0.03	23.17 ± 0.09	17.74 ± 0.01	13.97 ± 0.05
C18:1	Methyl Oleate	32.00 ± 0.17	31.07 ± 0.09	36.03 ± 0.50	32.96 ± 0.45
C20:1	Methyl cis-11-eicosenoate	0.01 ± 0.01	0.04 ± 0.02	0.17 ± 0.01	0.03 ± 0.01
C22:1	Methyl Erucate	5.13 ± 0.02	6.47 ± 0.18	5.52 ± 0.52	5.35 ± 0.02
C24:1	Methyl Nervonate	0.00 ± 0.00	0.21 ± 0.11	0.09 ± 0.04	0.05 ± 0.01
**Poly Unsaturated Fatty Acid (PUFA)**					
C18:2n-6	Methyl Linoleate	0.77 ± 0.05	3.25 ± 0.10	1.16 ± 0.06	2.05 ± 0.01
C20:3n-6	Methyl 11-14-17- Eicosatrienoate	0.44 ± 0.03	0.15 ± 0.06	1.22 ± 0.05	0.22 ± 0.09
C20:4n-6	Methyl Arachidonate	0.04 ± 0.01	0.05 ± 0.03	0.47 ± 0.00	0.10 ± 0.03
C18:3n-3	Methyl Linolenate	0.89 ± 0.03	0.25 ± 0.00	1.22 ± 0.01	1.23 ± 0.00
C20:5n-3	Methyl icosa-5,8,11, 14,17-pentaenoate	2.58 ± 0.05	1.13 ± 0.00	0.27 ± 0.04	2.60 ± 0.05
C22:5n-3	Methyl Docosapentaenoate	1.67 ± 0.47	0.80 ± 0.00	0.01 ± 0.00	1.75 ± 0.05
C22:6n-3	Methyl Docosahexanoate	0.33 ± 0.04	0.13 ± 0.00	0.15 ± 0.00	0.20 ± 0.00

Amino acid contents (% amino acid) are presented in the Table 4. Non-essential amino acids (61.12 ± 0.05 to 64.62 ± 0.03) are more prevalent than essential amino acids (35.38 ± 0.02 to 38.84 ± 0.04) in case of *Oscillatoria* spp.

**Table 4:** Amino acid content (% amino acids) of *Oscillatoria* spp. (mean ± SE). Here, **EAA**= Essential Amino Acid, **NEAA**= Non-Essential Amino Acid, **AA**= Total Amino Acid.

Compound Name (570 nm)	Code Name	Types	*Oscillatoria*			
			Species 1	Species 2	Species 3	Species 4
			
			Amount (%)			
Histidine	HIS	EAA	4.19 ± 0.01	0.11 ± 0.02	4.73 ± 0.02	3.24 ± 0.06
Isoleucine	ILE	EAA	2.83 ± 0.02	3.02 ± 0.02	2.06 ± 0.01	2.79 ± 0.02
Leucine	LEU	EAA	7.98 ± 0.03	7.71 ± 0.05	7.51 ± 0.01	7.63 ± 0.03
Lysine	LYS	EAA	4.54 ± 0.04	4.21 ± 0.02	6.07 ± 0.02	3.64 ± 0.04
Methionine	MET	EAA	1.85 ± 0.08	2.25 ± 0.00	2.14 ± 0.03	1.97 ± 0.02
Phenylalanine	PHE	EAA	3.36 ± ± 0.02	4.01 ± 0.04	3.86 ± 0.02	3.57 ± 0.02
Threonine	THR	EAA	5.40 ± 0.02	5.80 ± 0.10	5.20 ± 0.03	6.00 ± 0.04
Tyrosine	TYR	EAA	3.75 ± 0.02	4.06 ± 0.08	3.64 ± 0.02	4.03 ± 0.02
Valine	VAL	EAA	3.68 ± 0.02	4.22 ± 0.05	3.64 ± 0.01	3.87 ± 0.03
		**ƩEAA**	**37.58** ± **0.06^b^**	**35.38** ± **0.02^d^**	**38.84** ± **0.04^a^**	**36.72** ± **0.12**
Alanine	ALA	NEAA	12.65 ± 0.07	9.96 ± 0.04	12.50 ± 0.00	12.99 ± 0.05
Arginine	ARG	NEAA	7.05 ± 0.02	5.99 ± 0.02	6.25 ± 0.02	7.11 ± 0.02
Aspartic acid	ASP	NEAA	12.12 ± 0.02	13.33 ± 0.04	11.63 ± 0.02	13.21 ± 0.03
Glutamic acid	GLU	NEAA	15.06 ± 0.03	17.93 ± 0.05	13.93 ± 0.03	13.74 ± 0.05
Glycine	GLY	NEAA	5.70 ± 0.01	7.02 ± 0.03	6.45 ± 0.02	6.19 ± 0.06
Cysteine	CYS	NEAA	0.07 ± 0.01	1.73 ± 0.01	0.21 ± 0.00	0.14 ± 0.02
Serine	SER	NEAA	5.99 ± 0.02	5.97 ± 0.04	5.83 ± 0.00	6.28 ± 0.10
Proline	PRO	NEAA	3.74 ± 0.00	2.72 ± 0.03	4.34 ± 0.01	3.59 ± 0.00
		**ƩNEAA**	**62.37** ± **0.03^c^**	**64.62** ± **0.03^a^**	**61.12** ± **0.05^d^**	**63.23** ± **0.15^b^**
		**ƩAA/ƩEAA**	**2.66** ± **0.00^c^**	**2.83** ± **0.00^a^**	**2.57** ± **0.00^d^**	**2.72** ± **0.01^b^**
		**ƩAA/ƩNEAA**	**1.60** ± **0.00^b^**	**1.55** ± **0.00^d^**	**1.64** ± **0.00^a^**	**1.58** ± **0.00^c^**
		**ƩEAA/ƩNEAA**	**0.60** ± **0.00^b^**	**0.55** ± **0.00^d^**	**0.64** ± **0.00^a^**	**0.58** ± **0.00^c^**

## 4 Experimental Design, Materials and Methods

### 4.1 Cyanobacteria sampling site

Cyanobacterial samples were procured from both freshwater and marine environments, encompassing the collection of freshwater cyanobacteria samples from two locations in Chattogram, Bangladesh, comprising Kaptai Lake, Rangamati (22°64′ N, 92°19′ E) and Halda River, Chattogram (22°51′ N, 91°84′ E). Marine cyanobacteria were obtained from two stations in the Bay of Bengal including Maheshkhali channel, Cox's Bazar (21°31′ N, 91°59′ E) and Naf River estuary, Teknaf, Cox's Bazar (20° 47′ N, 92° 28′ E). Samples were collected between the months of February and July.

### 4.2 Physicochemical parameters

Physical parameters of the sampling site including temperature, DO and pH were measured with a glass thermometer, dissolved oxygen meter (DO-5509, Lutron) and a portable pH meter (pHep-HI98107, HANNA, Romania) respectively. Total ammonia nitrogen (TAN), Soluble reactive phosphorous (SRP) and Nitrite-nitrogen (NO_2__—_N) were determined spectrophotometrically according to Parsons et al. [5]. Three different standard solutions were prepared for analysis. TAN, SRP and NO_2__—_N of the sample water were measured at 640, 543 and 885 nm wavelength respectively through optical absorbance of the spectrophotometer (T80 UV/VIS Spectrophotometer).

### 4.3 Sample collection and concentration

Water sample was obtained by filtering about 40–50 L water through 60 µm mesh size plankton net, which was then collected in a 300 ml sample bottle and kept chilled while being transported to the laboratory. Then the samples were concentrated by centrifuging at 3000 rpm for 5 min. After centrifugation supernatant were discarded and the final concentrate was used for isolation.

### 4.4 Isolation of cyanobacteria

Liquid media Bold Basal Media (BBM) [6] was used for freshwater and Conway media [7] was used for marine cyanobacteria isolation. Liquid media were used followed the agar plate method (1.5% agar) for cyanobacteria isolation. Parallel streaking of concentrated sample was performed on the prepared agar plates by using nano loop. After 7–10 days of incubation, cyanobacterial growth was visible in the agar, then the petri dish was removed from the incubator and selected colony was placed on a glass slide with 1–2 drops of liquid media through a sterile nano loop. Then the colonies were microscopically observed to select the unialgal colonies that are free from any sort of contamination for further isolation. When the colony contained multiple microalgae, streak plate procedure was repeated until the pure single colony obtained.

### 4.5 Growth curve determination

The isolated cyanobacterial species were cultured utilizing the BBM and Conway medium for freshwater and marine isolates respectively. For growth curve experiment, three replicates of each species were prepared using sterile 500 mL borosilicate Erlenmeyer flasks, each containing a culture volume of 350 mL and inoculated with 2–3% pure culture stocks. The cultures were maintained under continuous light conditions of 24 h, with an intensity of 150 µEm-2s-^1^, gentle aeration at a rate of 4.53 ± 0.53 mg/L, and a temperature of 24 ± 1 °C. The experiment was carried out until the death phase and the growth curve was completed based on spectrophotometric analysis of chlorophyll content and optical density (absorbance).

#### 4.5.1 Determination of chlorophyll

##### 4.5.1.1 *Extraction of Oscillatoria* sp

Extraction of *Oscillatoria* sp. was performed using chemical method. To extract *Oscillatoria* spp. for chlorophyll content, 1 ml MgCO_3_ was filtered through a glass microfiber filter paper (47 mm Ø Whatman® GF/C) using a filter machine. Then, 1 ml of each *Oscillatoria* sp. sample and in case of water quality, 10 ml water sample was filtered. Following this, filter paper was two-folded and placed in a 15 ml centrifuge tube with the center facing downwards. Then, 2 ml of 90% acetone was added in the tube and homogenized for 1 min, 8 ml of 90% acetone are then added, and the mixture was then ground for 30 s. Followed this, the sample was refrigerated in the dark for 1 hour. After 1 hour, the sample was centrifuged at 3000 rpm for 10 min, and the acetone extract was transferred to another fresh centrifuge tube and centrifuged at low speed (500 rpm) for 5 min. Finally, the absorbance of the acetone extract was measured using 90% acetone as a blank.

##### 4.5.1.2 Chlorophyll quantification

Chlorophyll concentration was quantified based on spectrophotometric method [8]. The clear acetone extract was carefully transferred into a 1 cm cuvette and optical density (OD) was recorded at 750 nm, 664 nm, 647 nm, and 630 nm wavelengths. The OD values at 664 nm, 647 nm, and 630 nm were used to calculate chlorophyll concentration, while the OD value of 750 nm was used as turbidity correction factor and subtracted from each of the pigments OD values before using them in the equations. The concentrations of chlorophyll a, was calculated using the corrected OD values in the following equations [9]:

$$C_{a}=11.85\left(OD664\right)-1.54\left(OD647\right)-0.08\left(OD630\right)$$

Where: C_a_= Chlorophyll-a concentration in mg/L, and OD664, OD647, and OD630 = corrected optical densities (with a 1 cm light path) at the respective wavelengths. Once the pigment concentrations in the extract were determined, the pigments' quantity per unit volume was computed using the following formula:

$$Chlorophyll\;a\;\left(mg/m^{3}\right)=\frac{Ca\;\left(mg/L\right)\;\times extract\;volume\left(L\right)}{volume\;of\;sample\left(m^{3}\right)\;\;}$$

#### 4.5.2 Determination of optical density (OD)

To analyze the growth curve, optical density (OD) was measured daily, using respective culture media as the blank sample (BBM, Conway media). For each microalga, the maximum absorbance value was used to create the growth curve based on OD. The maximum absorbance was measured at specific wavelengths for each microalga, ranging from 443 nm to 600 nm, as those wavelengths showed the highest absorbance when scanned between 300 and 700 nm using a spectrophotometer (Nano Drop Spectrophotometer, Model-Nanoplus, Germany). Species 1 M1 (20) sp. showed maximum absorbance at 600 nm, Species 2 M2 (25) at 443 nm, Species 3 Fw1 at 530 nm, and Species 4 Fw2 at 475 nm.

### 4.6 Mass culture of microalgae

The large-scale or mass culture of selected microalgae isolates was conducted in tanks using both BBM and Conway medium respectively for freshwater and marine microalgae. The process involved gradually increasing the culture volume from an initial starter culture of 20 ml to 20 L. Initially, 20 ml microalgae stock were cultured in 30 ml of liquid medium in each flask, creating a total culture volume of 50 ml. The batch cultures were then incrementally scaled up to 100 ml, 250 ml, 500 ml, 1 L, and finally 10L-15 L, serving as inoculum for the subsequent step before transferring to a 20 L culture medium. PVC pipe substrates were used for Cyanobacteria culture. After reaching their stationary phase on day 12, microalgae species were harvested by centrifugation at 5500 rpm for 5 min by using centrifugation machine (TL5R Free Standing low speed refrigerated centrifuge, Herexi).

### 4.7 Preparation of dried biomass

The wet microalgae biomass obtained from post-centrifugation was subsequently oven-dried overnight at 40 °C. A high-quality hot air oven (JSR Korea's Natural Convention Oven LNO-150) was employed for drying, and the dried biomass was crushed into tiny particles (0.4–0.5 mm diameter) using a mortar and pestle. The powdered microalgae were then stored in a standard freezer at 4 °C until required for further use.

### 4.8 Nutritional compositions determination

#### 4.8.1 Protein determination

The protein content of each *Oscillatoria* sample was determined using the Lowry method [10]. Firstly, 5 mg of freeze-dried biomass was mixed with 25 mL of distilled water using a tissue homogenizer. Then, 0.5 mL of each sample was taken and mixed with 0.5 mL of 1 N NaOH, followed by heating in a hot water bath at 100 °C for 5 min. The samples were then cooled in a cold-water bath for 10 min. Then, 2.5 mL of a mixed reagent consisting of 50 mL of Reactive 2 (2 g of Na_2_CO_3_ in 100 mL of 0.1 NaOH) and 1 mL of Reactive 1 (1% NP tartrate) was added to ach sample. After proper mixing using vortex mixture, 0.5 mL of Folin reagent was added to each sample and the mixture was allowed to stand in the dark for 30 min. The absorbance was measured at 750 nm wavelength using a spectrophotometer. To create a calibration curve, a stock solution of albumin at 2000 µg/L was prepared and diluted to various concentrations (20 µg/L, 40 µg/L, 80 µg/L, 100 µg/L and 200 µg/L). The protein content of each sample was then determined using the standard curve obtained from the absorbance readings.

#### 4.8.2 Carbohydrate determination

The carbohydrate content of the *Oscillatoria* samples was determined using the method described by Dubois et al. (1956) [11]. Firstly, 5 mg of freeze-dried biomass was taken and mixed with 25 mL distilled water using a tissue homogenizer to prepare a well-mixed microalgae solution. From this solution, 1 mL was taken from each type of sample and mixed with 1 mL of 5% phenol and 5 mL of concentrated sulfuric acid (98%). The mixture was then left to react for 30 s and then cooled in a cold-water bath. The solution was then analyzed using spectrophotometric measurements at a wavelength of 488 nm. To prepare the calibration graph, 1000 µg/L standard glucose stock solution and a series of standards at various dilutions (20 µg/L, 40 µg/L, 60 µg/L, 100 µg/L, and 140 µg/L) were prepared. The same carbohydrate analysis procedure was applied to the standards as described above, and a standard graph was plotted according to the standard results. Using this graph, the carbohydrate content of each type of sample was determined based on the absorbance readings obtained.

#### 4.8.3 Lipid determination

The lipid content of the samples was determined according to the Bligh and Dyer [12] and Folch et al. (1957) [13] methods. Each sample was labelled and weighed in aluminum dishes to obtain the initial weight. 50 mg sample was taken in a centrifuge tube and diluted into 5x volume using distilled water. A solution of methanol: chloroform (2:1, v/v) was added, and the mixture was homogenized using a tissue homogenizer and centrifuged at 1000 rpm for 4 min at 4 °C. After centrifugation, the supernatants were transferred to clean tubes by Pasteur pipette and kept on ice. The remaining pellet was mixed with another solution of methanol: chloroform (2:1, v/v), centrifuged again under the same conditions, and combined with the previous supernatants. Then, 1.5 mL of 0.9% NaCl was added to the combined supernatants, and mixed thoroughly using a vortex mixer. The mixture was then refrigerated for 1 hour at 4 °C, followed by centrifugation at 1000 rpm for 10 min at 4 °C, resulting in the formation of two separate layers. The upper layer of methanol and chloroform was discarded, while the lower layer was transferred to a pre-prepared aluminum dish. The solvent was evaporated at 60 °C using a hot air oven, and the final weight of the aluminum dishes was determined to obtain the lipid weight in each sample by subtracting the initial weight from the final weight.

### 4.9 Fatty acid determination

Two steps transesterification also known as 2TE method with a little modification [14] was used to determine the fatty acid composition. In a lipid extraction beaker, 500 mg microalgae powder dissolved in 70 ml diethyl ether. Digital Soxhlet Apparatus (FOOD ALYTRD40) was used for lipid extraction. Diethyl ether was removed by placing the test tubes in the Hot Air Oven at 60 °C. Then, 1.5 ml of methanolic NaOH was added into the lipid extract and mixed properly through Sonication at 80 °C for 5 min. Upon cooling at room temperature (25 °C), 2 ml of BF_3_ methanol was poured into the mixture and again sonicated for 30 min at 80 °C. After cooling at 25 °C, 1 ml of isooctane and 5 ml of saturated NaCl was poured and well mixed through shaking. Then two layers were observed. Fatty acid methyl-esters (FAMEs), an organic substance in the upper layer was transferred to a new test tube. 1 ml sample from the test tube was taken into 1.5 ml Eppendorf vial for further fatty acid methyl-esters analysis through GCMS-Gas Chromatography Mass Spectrophotometry (GC-2020plus, SHIMADZU, Japan). Separation of FAMEs was done with a capillary column (30 m length, 0.25 mm internal diameter, 0.15 µm film thickness, and phase ratio is 250). Helium gas was used as a carrier gas with 1.42 ml/min flow rate. The column temperature program was: 180° to 280 °C at 5 °C /min and then at 280 °C. Detection of FAMEs were done by comparing the retention time with standard (FAME mix C8-C24; Sigma- Aldrich; Germany).

### 4.10 Amino acid determination

The Moore and Stein technique was slightly modified in order to identify amino acids [15]. 1 g dried biomass of microalgae was first hydrolyzed for 24 h at 110 ± 2 °C in 25 mL of previously prepared acidic hydrolysis solution (6 M HCl + 0.1% phenol). The samples were stabilized using a little quantity of SDB/Na (Sample Dilution Buffer) after cooling. The samples' pH was then adjusted using a basic neutralizing agent to range between 2.1 and 2.3. The hydrolysates were then filtered and diluted with SDB/Na before being put into the injection vials. SYKAM S 433 amino acid analyzer with UV detector was used for the analysis. With a constant flow rate of 0. 5 mL/min of nitrogen gas at a temperature of 60 °C and a reproducibility of 3%, nitrogen gas was employed as the carrier gas. Sigma-Aldrich, Germany's AA-S-18 standard wease is used to measure the concentration of amino acids. The amount of amino acids was measured in mg/g, which was then converted to% of all amino acids.

### 4.11 Statistical analysis

All sort of statistical analyses regarding the physicochemical water quality parameters, chlorophyll-a content, optical density, protein, carbohydrate, lipid content, fatty acid, amino acid contents were performed by using the IBM SPSS (v. 26.0). Descriptive statistics were performed for each of the cyanobacteria for each of the parameters; following that, test for homogeneity of variance was performed. Finally obtained data were analyzed by one-way analysis of variance (ANOVA) and significant differences amongst *Oscillatoria* species were analyzed using Tukey's multiple comparison tests at 95% confidence interval level. Post-hoc test was utilized to discern differences between groups.

## Limitations

Not applicable.

## Ethical Statement

No conflicts, informed consent, or human or animal rights are applicable to this study.

## CRediT authorship contribution statement

**Jannatul Nayeem:** Methodology, Data curation, Writing – original draft. **Proma Dey:** Data curation, Formal analysis. **Sumit Kanti Dey:** Data curation. **Rezaul Karim:** Data curation. **Mohammed Ayoun:** Data curation, Formal analysis. **Abed Hasan Tuser:** Data curation, Formal analysis. **Nusrat Zaman Zemi:** Data curation, Formal analysis. **Helena Khatoon:** Conceptualization, Funding acquisition, Supervision, Resources, Validation, Writing – review & editing.

## Data Availability

Dataset representing growth performance, nutritional assay and biochemical profile of Oscillatoria spp. (Original data) (Mendeley Data).
